# Reflective–dialogic classroom climate and adolescents’ academic purpose: a multilevel moderated mediation model

**DOI:** 10.3389/fpsyg.2026.1828786

**Published:** 2026-08-26

**Authors:** Ruo Jia, Xun Yin, Sidi Chu

**Affiliations:** School of Media Science (School of Journalism), Northeast Normal University, Changchun, China

**Keywords:** academic purpose, emotional regulation, metacognitive awareness, multilevel modeling, reflective–dialogic classroom climate

## Abstract

**Introduction:**

Understanding how classroom environments foster adolescents’ academic purpose—a future-oriented sense of meaning and direction in academic pursuits—remains a critical challenge in educational research. Although previous studies have linked classroom environments with immediate learning outcomes, relatively little is known about the multilevel mechanisms through which shared classroom climate **is** associated with adolescents’ academic purpose or the conditions under which these associations become stronger. Drawing on self-regulated learning and socio-ecological perspectives, this study examines a multilevel model in which reflective–dialogic classroom climate is conceptualized as a classroom-level resource associated with students’ academic purpose through individual self-regulatory mechanisms.

**Methods:**

Using data collected from adolescents nested within classrooms, multilevel structural equation modeling was conducted in Mplus to test a dual-stage moderated mediation framework.

**Results:**

Reflective–dialogic classroom climate was positively associated with metacognitive awareness (*β* = 0.32, *p* < 0.001), which, in turn, was positively associated with academic purpose (*β* = 0.38, *p* < 0.001). Emotional regulation moderated both the association between classroom climate and metacognitive awareness (*β* = 0.14, *p* = 0.001) and the association between metacognitive awareness and academic purpose (*β* = 0.12, *p* < 0.001). Conditional indirect effects further indicated that the indirect association between classroom climate and academic purpose through metacognitive awareness was stronger among students with higher emotional regulation.

**Discussion:**

The findings provide a multilevel perspective on how classroom-level contexts and individual self-regulatory capacities are associated with adolescents’ academic purpose, thereby enriching the application of self-regulated learning theory in educational settings and offering practical implications for designing reflective learning environments.

## 1 Introduction

Adolescence represents a critical developmental period during which students begin to form enduring beliefs about the meaning, direction, and value of their academic experiences. Beyond short-term engagement or performance outcomes, scholars have increasingly emphasized the importance of academic purpose as a motivational orientation that anchors learning in long-term goals and personal meaning (Wang et al., 2022). In the present study, academic purpose refers specifically to students’ sense that their academic activities are meaningful, personally significant, and connected to future-oriented educational goals; it is therefore distinct from the broader construct of purpose in life and from academic motivation, which primarily concerns the direction, intensity, and persistence of learning behavior. A clear sense of academic purpose has been linked to sustained effort, resilience in the face of challenges, and positive educational trajectories across adolescence (Guthrie et al., 2021; Harefa et al., 2023). Despite its recognized importance, relatively little is known about how educational contexts are associated with the development of academic purpose, particularly during secondary schooling where motivational disengagement often intensifies.

Recent advances in educational research have highlighted that students’ motivational orientations do not emerge in isolation but develop in conjunction with ongoing interactions with instructional environments. Classroom climates that encourage dialogue, reflection, and shared meaning-making have been shown to support deeper learning and engagement by positioning students as active contributors to the learning process rather than passive recipients of information (Brestovanský and Sopoliga, 2025). Such environments provide opportunities for students to articulate reasoning, consider alternative perspectives, and reflect on their learning experiences, all of which are foundational for developing higher-order cognitive and motivational capacities. However, much of the existing literature has focused on immediate academic outcomes, such as engagement (Vasalampi et al., 2021) or achievement (Tong and Ding, 2025), leaving open questions about whether and how reflective and dialogic classroom environments are associated with more enduring motivational constructs such as academic purpose.

Zimmerman’s self-regulated learning theory provides the primary conceptual framework for explaining these relationships. Self-regulated learning is understood as a cyclical process through which students proactively regulate their cognition, motivation, behavior, and affect in relation to personally valued academic goals (Zimmerman, 2013). From this perspective, instructional environments do not directly determine students’ motivational outcomes; rather, they provide social and instructional cues that may become consequential through students’ internal self-regulatory processes. Prior research has consistently demonstrated that learners who are able to monitor, evaluate, and regulate their thinking processes are better equipped to engage meaningfully with academic tasks and to align learning activities with long-term goals (Chandra Handa, 2023). Metacognitive awareness, in particular, has been identified as a central mechanism through which students translate instructional cues into purposeful learning behavior (Stewart and Reinders, 2023). Reflective–dialogic classroom practices may be especially relevant to this process because they repeatedly invite students to explain their reasoning, assess their understanding, reconsider ineffective strategies, and evaluate their learning progress. While classroom practices that promote reflection and dialogue are assumed to stimulate metacognitive processes, empirical work explicitly examining this mediating pathway remains limited, especially in adolescent populations.

In parallel, growing evidence suggests that cognitive regulation alone is insufficient to explain how students derive meaning from their academic experiences. Emotional processes play a critical role in students’ engagement with reflective and cognitively demanding learning environments. In this regard, De Neve et al. (2023) suggest that emotional regulation is associated with how students respond to academic challenges, manage stress, and sustain motivation over time. This is further substantiated by Vistorte et al. (2024), who argued that learners with stronger emotional regulation skills are more likely to persist in complex tasks, reframe difficulties constructively, and benefit from cognitively demanding instructional practices. Within an SRL framework (Zimmerman, 2013), emotional regulation may help students maintain the attentional and motivational resources needed to respond metacognitively to reflective classroom practices and to connect such metacognitive awareness with a sustained sense of academic purpose. Yet, emotional regulation has rarely been examined as a boundary condition that may qualify both the activation of metacognitive processes and their motivational consequences, resulting in an incomplete understanding of how cognition and emotion jointly operate within classroom contexts.

An additional limitation of prior research concerns the predominant reliance on single-level analytical approaches. Although classroom environments are inherently shared experiences, many studies treat instructional climate as an individual perception rather than a collective property of classrooms. Multilevel theory and methodology emphasize that failing to account for the nested structure of educational data may obscure contextual effects and lead to biased conclusions about learning processes (Granziera et al., 2023). Conceptualizing classroom climate at the group level allows for a more accurate examination of how shared instructional practices are associated with individual self-regulatory processes and motivational outcomes, while simultaneously accounting for individual differences among students. A multilevel approach is therefore necessary because it distinguishes variation attributable to the shared classroom context from variation attributable to individual students. However, a multilevel direct-effects model alone would not explain the psychological process underlying this contextual association or why students within the same classroom may respond differently. A multilevel moderated mediation model is consequently required to examine whether metacognitive awareness statistically accounts for the association between classroom-level climate and academic purpose and whether emotional regulation conditions both stages of this indirect relationship.

Taken together, three interrelated gaps remain in the literature. First, previous research has primarily connected reflective or dialogic classroom environments with proximal outcomes, such as engagement and achievement (Vasalampi et al., 2021; Tong and Ding, 2025), while devoting less attention to academic purpose as a distinct, future-oriented motivational orientation. Second, classroom climate, metacognitive awareness, and emotional regulation have commonly been examined through separate or direct-effect models, leaving the mediating role of metacognitive awareness and the dual-stage moderating role of emotional regulation insufficiently understood (Stewart and Reinders, 2023; De Neve et al., 2023; Vistorte et al., 2024). Recent evidence likewise suggests that educational outcomes among Chinese learners are better explained through multifactorial frameworks that simultaneously consider cognitive and contextual influences (Zhang and Chen, 2026). Third, prior studies have often treated classroom climate as an individual-level perception, thereby overlooking its shared contextual nature and the nested structure of educational data (Granziera et al., 2023). These limitations prevent a comprehensive understanding of how classroom-level instructional conditions and individual regulatory capacities are jointly associated with adolescents’ academic purpose.

Addressing these gaps, the present study adopts a multilevel perspective to examine how reflective–dialogic classroom climate is associated with adolescents’ academic purpose through internal self-regulatory mechanisms. Building primarily on self-regulated learning theory (Zimmerman, 2013), and supported by socio-ecological perspectives that recognize classrooms as shared developmental contexts (Allen and Vella-Brodrick, 2018), the study proposes that classroom-level instructional climate is indirectly associated with academic purpose through metacognitive awareness. Moreover, drawing on emotion regulation frameworks (Werner and Gross, 2010), emotional regulation is proposed as a critical boundary condition that may moderate both the extent to which students respond to classroom-level learning resources and the degree to which metacognitive awareness is associated with academic purpose. By testing a dual-stage moderated mediation model using multilevel structural equation modeling, this study examines the joint operation of classroom-level and individual-level processes while avoiding the conflation of shared contextual effects with individual perceptions.

The study makes three principal contributions that distinguish it from prior research. Theoretically, it extends self-regulated learning theory by integrating classroom-level instructional climate with individual self-regulatory processes to explain adolescents’ academic purpose within a single multilevel framework. It further incorporates emotional regulation as a dual-stage boundary condition, thereby explaining why students exposed to similar classroom environments may differ in both their metacognitive responses and the strength of the association between metacognitive awareness and academic purpose. Methodologically, the study conceptualizes reflective–dialogic classroom climate as a Level-2 construct and tests the proposed conditional indirect associations through multilevel structural equation modeling. Because the study uses cross-sectional data, the proposed pathways are interpreted as theoretically ordered associations rather than evidence of temporal precedence or causal effects.

## 2 Literature

### 2.1 Hypothesizing reflective-dialogic classroom climate and metacognitive awareness

The study anticipates that instructional environments are associated with differences in how students become aware of and regulate their own learning processes. Zimmerman’s (2013) self-regulated learning perspective suggests that metacognitive awareness develops when learners repeatedly engage in planning, monitoring, and evaluating their cognition. From this perspective, reflective–dialogic classroom climates provide external regulatory cues that encourage students to inspect their reasoning, assess their understanding, and reconsider the strategies used during learning. Through repeated exposure, these socially provided prompts may gradually be internalized as students’ own metacognitive routines. Classrooms that emphasize dialogue, explanation, and reflection require students to articulate reasoning, evaluate understanding, and reconsider ideas through interaction, all of which are central to metacognitive awareness (Bae and Kwon, 2021). Such environments shift attention away from surface task completion toward ongoing monitoring of cognition, thereby creating conditions that are conducive to the development of metacognitive awareness.

In addition, a growing body of empirical research demonstrates that reflective and dialogic instructional practices are closely associated with students’ metacognitive functioning. For instance, Fomin (2024) showed that classrooms encouraging explanation and reflection were more likely to elicit students’ planning and monitoring behaviors during learning. This pattern is further endorsed by Cui and Teo (2021), who reported that discussion-oriented classroom practices were positively related to adolescents’ use of metacognitive learning strategies. Similarly, large-scale syntheses indicate that instructional approaches making thinking visible—such as peer explanation, questioning, and reflective dialogue—are positively associated with students’ metacognitive development (Nobutoshi, 2023).

Beyond individual instructional techniques, the broader classroom climate appears particularly consequential. Tong and Ding (2025) argue that when reflection and dialogue become normative features of classroom interaction, students are more likely to internalize regulatory questions concerning understanding and strategy use. Moreover, evidence suggests that such climates are especially important during adolescence, a period marked by increasing cognitive complexity alongside uneven self-regulatory capacity, because external dialogic prompts may provide scaffolding for emerging internal metacognitive control (Brestovanský and Sopoliga, 2025).

Accordingly, reflective–dialogic classroom climate is not expected to determine metacognitive awareness directly. Rather, it constitutes a shared instructional context in which regulatory questioning, explanation, and evaluation are repeatedly modeled and practiced. Students exposed to such a climate may therefore become more attentive to what they understand, how they approach academic tasks, and when their learning strategies require adjustment. From a self-regulated learning perspective, these empirical patterns can be theoretically anchored in the assumption that metacognitive awareness develops through sustained engagement with environments that repeatedly prompt learners to plan, monitor, and evaluate cognition (Paethrangsi et al., 2024). Reflective–dialogic classroom climates function as contextual affordances that embed regulatory cues within everyday instructional interaction, supporting the gradual internalization of metacognitive processes.

Taken together, converging empirical findings and theoretical reasoning suggest that classrooms characterized by reflective and dialogic practices should be positively associated with students’ metacognitive awareness.

*Hypothesis 1*: Reflective–dialogic classroom climate is positively associated with students’ metacognitive awareness.

### 2.2 Hypothesizing metacognitive awareness and academic purpose

From the perspective of self-regulated learning theory, purposeful engagement in learning emerges when students actively coordinate cognitive regulation with personally valued academic goals (Zimmerman, 2013). Metacognitive awareness enables learners to monitor their understanding, evaluate progress, and adjust learning strategies in ways that align immediate academic tasks with broader educational aspirations. Rather than viewing learning as a series of isolated activities, students with greater metacognitive awareness are more likely to organize their academic experiences around coherent long-term objectives, thereby strengthening their sense of academic purpose. A growing body of research has linked students’ capacity to regulate and reflect on their learning processes with deeper motivational orientations toward education. Empirical studies consistently show that students who actively monitor their understanding, evaluate learning progress, and adjust strategies are more likely to perceive their academic activities as meaningful and aligned with longer-term goals (Bae and Kwon, 2021; Stewart and Reinders, 2023). Such learners tend to engage with academic tasks not merely as requirements but as opportunities for growth, which is a defining feature of academic purpose.

In addition, evidence suggests that metacognitive awareness supports students’ ability to connect immediate learning experiences with broader academic aspirations. For example, students who frequently reflect on how and why they learn have been found to demonstrate stronger goal clarity, persistence, and commitment to educational pursuits (Goren and Kaya, 2023). This relationship is further endorsed by studies showing that metacognitive regulation enhances students’ capacity to derive meaning from academic challenges, particularly in contexts requiring sustained effort and self-direction (Stanton et al., 2021; Yunus et al., 2021).

Moreover, during adolescence, when motivational orientations are especially malleable, metacognitive awareness appears to play a critical role in transforming academic demands into personally meaningful goals. Prior research indicates that adolescents who engage in reflective thinking about their learning processes are more likely to articulate future-oriented academic intentions and maintain a sense of direction despite setbacks (Breland et al., 2023; Tay et al., 2024). This suggests that metacognitive awareness functions not only as a cognitive resource but also as a motivational mechanism that supports the development of academic purpose.

The observed empirical relationships are consistent with self-regulated learning theory, which proposes that purposeful learning develops when learners integrate cognitive regulation with personally valued goals (Zimmerman, 2013). Within this framework, metacognitive awareness provides the regulatory mechanism through which students connect day-to-day learning activities with broader academic intentions and future aspirations. Accordingly, students who more effectively monitor, evaluate, and adapt their learning processes should be better positioned to perceive their academic experiences as meaningful and purpose-driven.

Taken together, converging empirical evidence and theoretical reasoning suggest that higher levels of metacognitive awareness should be positively associated with a stronger sense of academic purpose among students.

*Hypothesis 2*: Metacognitive awareness is positively associated with students’ academic purpose.

### 2.3 Hypothesizing the mediating role of metacognitive awareness

Building on the proposed associations between reflective–dialogic classroom climate and metacognitive awareness (Hypothesis 1), and between metacognitive awareness and academic purpose (Hypothesis 2), self-regulated learning theory provides a basis for viewing metacognitive awareness as the intervening regulatory mechanism connecting classroom context with students’ sense of academic meaning. Reflective and dialogic classroom practices expose students to recurring prompts to explain, monitor, and evaluate their learning; however, these contextual cues are unlikely to correspond with academic purpose unless students cognitively interpret and incorporate them into their own regulatory processes. Metacognitive awareness may perform this function by enabling students to connect immediate classroom experiences with broader academic goals and personally meaningful educational intentions. Prior research suggests that instructional environments are not always directly associated with motivational outcomes (Boyd et al., 2022; Chung, 2023; Gülşen and Atay, 2022); rather, their associations may be accounted for by students’ internal regulatory processes that shape how learning experiences are interpreted and valued.

In this regard, several studies have identified metacognitive awareness as a central explanatory process linking instructional practices and learning environments with higher-order motivational and attitudinal outcomes. For instance, empirical evidence indicates that classroom practices emphasizing reflection and cognitive articulation are positively associated with students’ metacognitive regulation, which, in turn, is associated with deeper goal orientation, persistence, and meaning in learning activities (Barak, 2024; Cui and Teo, 2021). This mediating role is further supported by research suggesting that metacognitive awareness helps students interpret contextual learning cues in relation to purposeful academic engagement by enabling them to align immediate tasks with broader academic intentions (Bae and Kwon, 2021; Breland et al., 2023).

Accordingly, reflective–dialogic classroom climate may be indirectly associated with academic purpose because its recurring regulatory cues correspond with greater metacognitive awareness, which is subsequently associated with students’ capacity to organize academic experiences around meaningful goals (Zimmerman, 2013; Akcaoğlu et al., 2023). This indirect pathway does not imply that classroom climate causally produces academic purpose; rather, it specifies the theoretically ordered process through which the association between a shared instructional context and an individual motivational orientation may be statistically accounted for.

*Hypothesis 3*: Metacognitive awareness mediates the relationship between reflective–dialogic classroom climate and students’ academic purpose.

### 2.4 Hypothesizing the moderating role of emotional regulation

Within self-regulated learning theory, effective learning depends not only on cognitive regulation but also on learners’ capacity to regulate emotional experiences that accompany challenging academic tasks (Zimmerman, 2013). Reflective–dialogic classroom environments frequently require students to justify ideas, reconsider misconceptions, respond to feedback, and tolerate uncertainty. These cognitively demanding situations often evoke emotional reactions that may either facilitate or constrain students’ engagement with reflective learning opportunities. Accordingly, emotional regulation represents an important self-regulatory resource that may determine the extent to which students benefit from classroom environments designed to promote reflection and dialogue. Although reflective–dialogic classroom environments and metacognitive awareness have been shown to support purposeful learning, accumulating evidence suggests that such benefits are not uniformly experienced by all students. Learners differ substantially in their capacity to manage emotional demands associated with cognitively challenging and socially interactive instructional contexts, which may shape how effectively they capitalize on reflective learning opportunities (De Neve et al., 2023). Emotional regulation has therefore been increasingly recognized as a critical boundary condition that conditions the effectiveness of both contextual and cognitive learning processes. Huang et al.’s (2025) study also indicates that students’ broader self-regulatory capacities can qualify the extent to which contextual support is associated with adaptive learning outcomes.

In educational settings, emotional regulation has been shown to influence how students respond to academic challenge, social evaluation, and uncertainty inherent in reflective and dialogic learning activities (Tu et al., 2026; Usán Supervía and Quílez Robres, 2021). In this regard, students with stronger emotional regulation skills are better able to tolerate cognitive dissonance, manage frustration, and remain engaged during demanding instructional interactions, whereas students with weaker regulation capacities may disengage or rely on surface-level strategies when faced with similar conditions (Vistorte et al., 2024). This is further supported by empirical studies indicating that emotional regulation enhances students’ capacity to benefit from cognitively demanding instruction by sustaining effort and adaptive engagement under emotional strain (Chu et al., 2025).

Although the present study conceptualizes emotional regulation as a moderator, its relationship with metacognitive awareness may be reciprocal. Students who effectively regulate frustration, anxiety, or uncertainty are generally better able to sustain attention and monitor their thinking during demanding learning activities. Conversely, greater metacognitive awareness may help students recognize emotional reactions, evaluate their potential influence on learning, and deliberately select appropriate regulatory strategies. Thus, cognition and emotion are best viewed as mutually reinforcing components of self-regulated learning rather than entirely independent processes (Zimmerman, 2013; De Neve et al., 2023). Nevertheless, because the purpose of the present study is to examine when reflective–dialogic classroom climate is more strongly associated with metacognitive awareness and academic purpose, emotional regulation is modeled as a boundary condition rather than as a reciprocal outcome. Given the cross-sectional design, this specification reflects a theoretically derived analytical ordering and should not be interpreted as establishing temporal or causal precedence.

Moreover, prior research suggests that emotional regulation moderates the extent to which instructional cues are internalized into higher-order cognitive processes. For example, learners with stronger emotional regulation are more likely to translate external instructional prompts into metacognitive awareness, as they can remain cognitively focused while managing affective responses elicited by reflective dialogue and peer interaction (De Neve et al., 2023; Usán Supervía and Quílez Robres, 2021; Vistorte et al., 2024). In parallel, evidence also indicates that emotional regulation conditions the motivational consequences of metacognitive processes, such that metacognitive awareness is more likely to foster academic meaning and goal alignment when students can effectively regulate emotions associated with effort, failure, or uncertainty (Merkebu et al., 2023).

Taken together, self-regulated learning theory and existing empirical evidence suggest that emotional regulation may strengthen students’ capacity to benefit from reflective–dialogic classroom environments while also enhancing the extent to which metacognitive awareness corresponds with academic purpose (Zimmerman, 2013). Accordingly, emotional regulation is expected to function as a dual-stage moderator, conditioning both the association between reflective–dialogic classroom climate and metacognitive awareness and the association between metacognitive awareness and academic purpose.

*Hypothesis 4a*: Emotional regulation moderates the relationship between reflective–dialogic classroom climate and metacognitive awareness, such that the relationship is stronger for students with higher levels of emotional regulation.

*Hypothesis 4b*: Emotional regulation moderates the relationship between metacognitive awareness and students’ academic purpose, such that the relationship is stronger for students with higher levels of emotional regulation.

### 2.5 Hypothesizing the moderated mediating role of emotional regulation

The proposed model further suggests that the indirect association between reflective–dialogic classroom climate and academic purpose through metacognitive awareness should vary according to students’ emotional regulation. From a self-regulated learning perspective (Zimmerman, 2013), cognitive and emotional regulatory processes operate jointly during learning, implying that the strength of an indirect association may depend on students’ capacity to regulate the emotional demands accompanying reflective learning activities (Zimmerman, 2013). Consequently, the mediating role of metacognitive awareness is expected to be stronger when students possess greater emotional regulation because they are better able to internalize classroom-level regulatory cues and translate metacognitive awareness into meaningful academic goals. As established earlier, classroom climate is positively associated with metacognitive awareness (Hypothesis 1), which, in turn, is positively associated with academic purpose (Hypotheses 2 and 3), and both of these associations are expected to vary according to emotional regulation (Hypotheses 4a and 4b). This expectation is also consistent with moderated mediation theory, which proposes that the magnitude of an indirect association depends on the level of a moderator operating on one or more constituent paths (Edwards and Lambert, 2007; Hayes, 2015).

Accordingly, emotional regulation is expected to condition the overall indirect association between reflective–dialogic classroom climate and academic purpose through metacognitive awareness, giving rise to a dual-stage moderated mediation process. Consistent with the study’s cross-sectional design, this conditional indirect effect is interpreted as a theoretically specified pattern of associations rather than evidence of a causal process.

**Figure 1:**
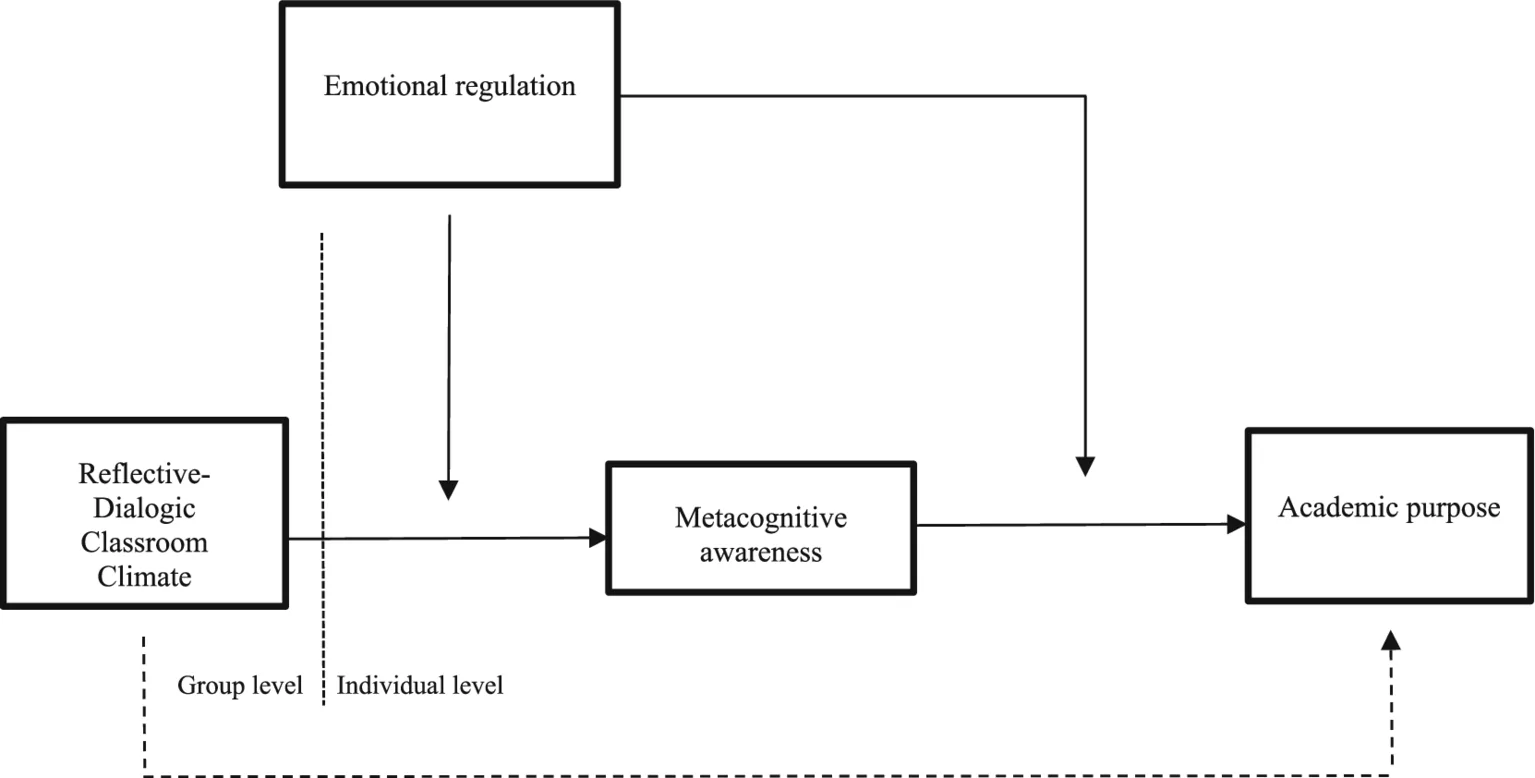
Proposed conceptual model.

*Hypothesis 5*: Emotional regulation moderates the indirect relationship between reflective–dialogic classroom climate and students’ academic purpose through metacognitive awareness, such that the indirect effect is stronger at higher levels of emotional regulation (Figure 1).

## 3 Methodology

**Figure 2:**
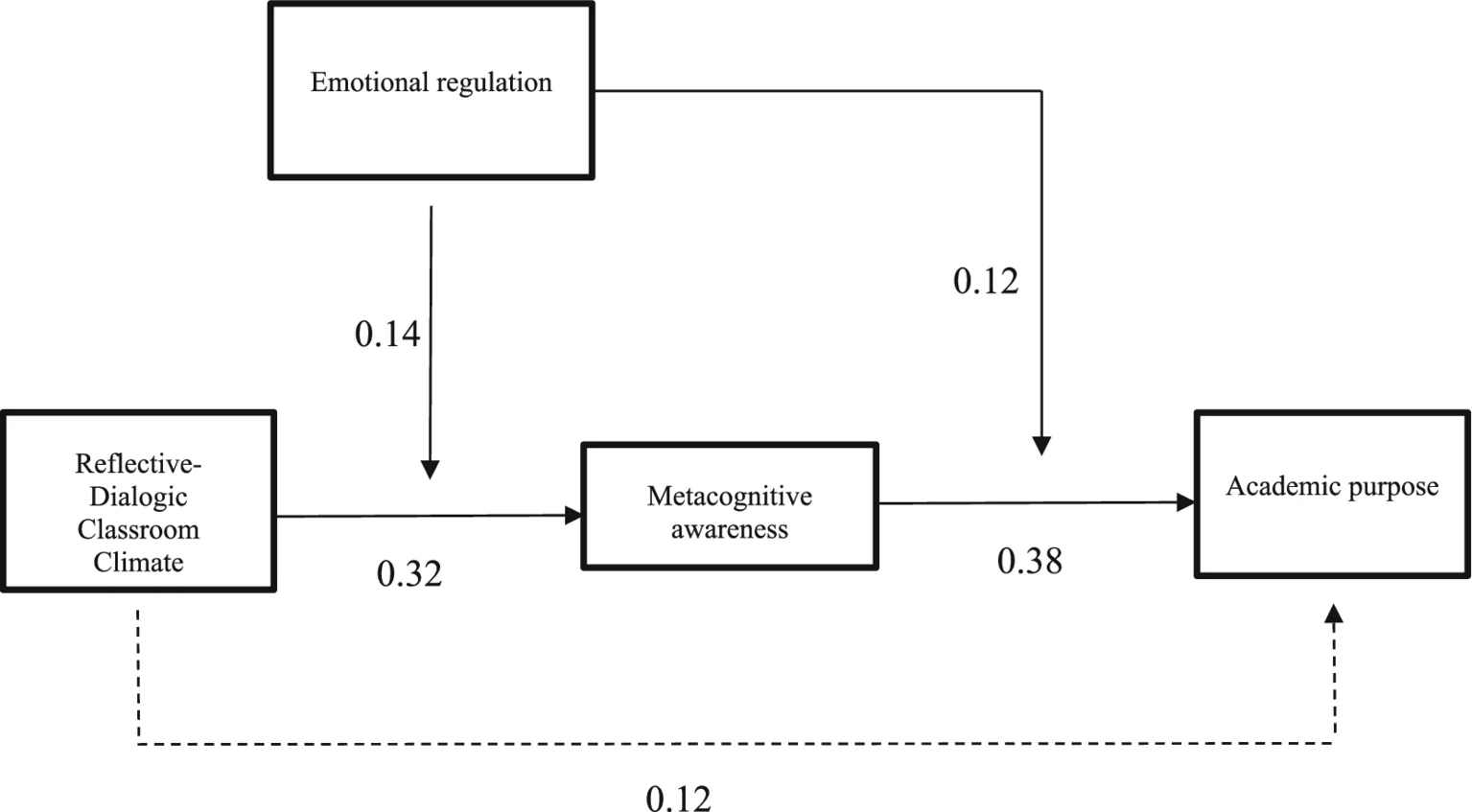
Structural path model.

The study employed a quantitative, cross-sectional research design grounded in a multilevel analytical framework to examine relationships among classroom-level and individual-level variables. Data were collected through an in-person survey administered to adolescent students enrolled in secondary schools in China. The school setting was considered appropriate for investigating reflective–dialogic classroom climate and self-regulatory processes, as classroom interactions and instructional practices are central to adolescents’ academic and motivational development within this context (Figure 2).

A probability-based, stratified multistage random sampling strategy was adopted to improve the representativeness of the participating schools and classrooms. Specifically, public secondary schools were first stratified according to locality (urban and suburban), as educational contexts in China may differ across geographical settings, making stratification appropriate for enhancing sample representativeness (Li et al., 2021). Within the participating schools, intact classrooms were then randomly selected, and all eligible students in the selected classrooms were invited to participate. A total of eight public secondary schools (five urban and three suburban) and 32 classrooms were included in the study. This probability-based approach was considered appropriate for multilevel research because systematic selection of naturally occurring classroom clusters improves the estimation of both within-classroom and between-classroom variance while reducing selection bias.

A total of 600 questionnaires were distributed in person across the selected classrooms with the support of school administrators and teachers. In-person administration was chosen to maximize response rates, ensure standardized instructions, and reduce missing data. After excluding incomplete and unusable responses, 512 valid questionnaires were retained for analysis, yielding an effective response rate of approximately 85%. The final analytical sample comprised 512 students nested within 32 classrooms, with classroom sizes ranging from 12 to 20 students (M = 16.00). The Level-2 sample size satisfied commonly recommended guidelines for multilevel structural equation modeling and provided adequate statistical power for estimating cross-level relationships. The estimated design effect further supported the use of a multilevel analytical framework.

The demographic profile of the respondents is summarized in Table 1. Approximately 52% of the participants were male and 48% were female. The average age of the students was 15.3 years, with ages ranging from early to late adolescence. In terms of grade level, about 38% of the students were enrolled in lower secondary grades, 42% in middle secondary grades, and the remaining 20% in upper secondary grades. With respect to location, roughly 65% of the respondents attended schools in urban areas, while 35% were from suburban settings. These demographic characteristics were included as control variables in the analysis to account for potential confounding effects.

**Table 1:** Sample and cluster characteristics.

Category	Characteristic	n	% / Statistic
Sampling structure	Participating schools	8	—
	Urban schools	5	62.5
	Suburban schools	3	37.5
	Participating classrooms (Level 2)	32	—
	Students retained (Level 1)	512	—
Data collection	Questionnaires distributed	600	—
	Questionnaires excluded	88	14.7
	Valid questionnaires	512	85.3
Cluster characteristics	Mean classroom size	—	16.00
	Classroom-size range	—	12–20
	Estimated design effect	—	1.95
Gender	Male	266	52.0
	Female	246	48.0
Age	Mean age	—	15.30 years
	Age range	—	13–18 years
Grade level	Lower secondary	195	38.1
	Middle secondary	215	42.0
	Upper secondary	102	19.9
School locality	Urban	333	65.0
	Suburban	179	35.0

Level 1 represents individual students and Level 2 represents classrooms.

Participation in the study was voluntary, and students were informed of the academic purpose of the research prior to data collection. Anonymity and confidentiality were assured, and all responses were used solely for research purposes. Written informed consent was obtained from the participating students and their parents or legal guardians before data collection. The study protocol was reviewed and approved by the Institutional Research Ethics Committee (Approval No. 2026001). Data collection procedures complied with institutional and ethical guidelines applicable to educational research involving adolescent participants.

Table 1 summarizes the sample and cluster characteristics. The data were collected from 512 students nested within 32 classrooms across eight secondary schools, providing an appropriate hierarchical structure for multilevel analysis. The sample was well distributed across gender, grade levels, and school locality, supporting the representativeness of the study sample.

## 4 Measures

All measurement instruments were adapted from previously validated scales to ensure their suitability for Chinese secondary school students. Following standard cross-cultural adaptation procedures (Beaton et al., 2000; Brislin, 1980), the original English items were translated into Chinese by two bilingual researchers and independently back-translated into English by a separate bilingual translator. Discrepancies were discussed until semantic equivalence was achieved. The translated questionnaire was subsequently reviewed by two educational psychology experts to assess content validity and age appropriateness. A pilot test involving 30 secondary school students confirmed that the items were clear and comprehensible, and only minor wording refinements were made before the main data collection.

### 4.1 Reflective–dialogic classroom climate

Reflective–dialogic classroom climate was measured using a six-item scale assessing students’ perceptions of classroom practices that emphasize reflective learning and dialogic interaction. Items were adapted from prior measures of dialogic teaching and reflective classroom environments (Applebee et al., 2003; Reznitskaya et al., 2007). A sample item is “In this class, students are encouraged to explain their thinking to others.” Responses were recorded on a five-point Likert scale (1 = strongly disagree, 5 = strongly agree). Individual responses were aggregated to the classroom level following established multilevel procedures.

### 4.2 Metacognitive awareness

Metacognitive awareness was assessed using an eight-item adapted version of the Metacognitive Awareness Inventory (Schraw and Dennison, 1994), capturing students’ awareness and regulation of cognitive processes during learning. A sample item is “I know when I understand a topic and when I do not.” Responses were provided on a five-point Likert scale (1 = strongly disagree, 5 = strongly agree).

### 4.3 Emotional regulation

Emotional regulation was measured using a six-item youth-adapted version of the Emotion Regulation Questionnaire (Gross and John, 2003), assessing students’ ability to manage emotional responses in academic contexts. A sample item is “I can calm myself when I feel upset about schoolwork.” Items were rated on a five-point Likert scale.

### 4.4 Academic purpose

Academic purpose was measured using a five-item scale adapted from established purpose and meaning measures for adolescents (Damon et al., 2003). The scale reflects students’ sense of direction and meaning in their academic pursuits. A sample item is “What I learn in school is meaningful for my future.” Responses were recorded on a five-point Likert scale.

## 5 Data analysis

The hierarchical structure of the data, with students nested within classrooms, warranted the use of multilevel modeling to account for non-independence of observations and to appropriately partition variance across levels (Raudenbush and Bryk, 2002; Hox et al., 2017). The proposed indirect and conditional indirect associations were evaluated within a multilevel structural equation modeling framework, which permits the simultaneous estimation of within- and between-level mediation processes (Preacher et al., 2010). Unconditional (null) models were first estimated to examine the proportion of variance attributable to between-classroom differences. Intraclass correlation coefficients [ICC (1)] were calculated to determine whether sufficient between-classroom variance existed to justify multilevel analysis.

The aggregation of reflective–dialogic classroom climate to the classroom level was evaluated using established indices of within-group agreement and reliability. Specifically, ICC (1) was used to assess the extent of between-classroom variability, ICC (2) was examined to determine the reliability of classroom-level means, and within-group agreement was assessed using rwg. The design effect was also calculated to further assess the influence of the nested data structure. These indices were evaluated against established methodological recommendations (Bliese, 2000; LeBreton and Senter, 2008).

All individual-level predictors were group-mean centered, whereas the classroom-level predictor was grand-mean centered, following established guidelines for multilevel models involving cross-level effects (Enders and Tofighi, 2007). Models were estimated in Mplus using maximum likelihood with robust standard errors, which provides estimation procedures suitable for structural models involving nested observations and cross-level relationships (Muthén and Muthén, 2017). The final multilevel structural equation model simultaneously estimated within-classroom and between-classroom structural relationships, specifying reflective–dialogic classroom climate as a Level-2 predictor and metacognitive awareness, emotional regulation, and academic purpose as Level-1 constructs. The model further incorporated cross-level moderation and conditional indirect associations within a single analytical framework, while indirect associations were evaluated using bias-corrected bootstrapped 95% confidence intervals based on 5,000 bootstrap resamples. Prior to hypothesis testing, common method bias, descriptive statistics, measurement reliability, convergent and discriminant validity, and confirmatory factor analysis were evaluated.

## 6 Results

Table 2 reports the aggregation statistics used to justify multilevel modeling. Reflective–dialogic classroom climate demonstrated an ICC (1) of 0.18, an ICC (2) of 0.78, an rwg of 0.91, and a design effect of 1.95. Collectively, these values indicate meaningful between-classroom variability, acceptable reliability of classroom-level means, and strong within-classroom agreement, thereby supporting aggregation of reflective–dialogic classroom climate to the classroom level and the use of multilevel modeling.

**Table 2:** Aggregation statistics and multilevel justification.

Classroom-level construct	ICC (1)	ICC (2)	rwg	Design effect	Aggregation decision
Reflective–dialogic classroom climate	0.18	0.78	0.91	1.95	Supported

ICC (1) = intraclass correlation coefficient (individual-level agreement attributable to classroom membership); ICC (2) = reliability of classroom means; rwg = within-group interrater agreement.

Table 3 presents the assessment of common method bias. Harman’s single-factor test indicated that the first unrotated factor accounted for 32.84% of the total variance, which is below the recommended threshold of 50%. Furthermore, the full-collinearity variance inflation factor (VIF) values ranged from 1.78 to 2.16, remaining well below the recommended cut-off value of 3.30. Collectively, these findings suggest that common method bias was unlikely to pose a serious threat to the validity of the results.

**Table 3:** Common method biasness assessment.

Assessment	Result	Conclusion
Harman’s single-factor test	32.84%	No substantial common method bias
Full collinearity VIF (reflective–dialogic classroom climate)	1.78	Acceptable
Full collinearity VIF (metacognitive awareness)	2.04	Acceptable
Full collinearity VIF (emotional regulation)	1.91	Acceptable
Full collinearity VIF (academic purpose)	2.16	Acceptable

VIF, variance inflation factor.

Table 4 presents the descriptive statistics and bivariate correlations among the study variables. The mean values indicate that students reported moderate to relatively high levels of reflective–dialogic classroom climate, metacognitive awareness, emotional regulation, and academic purpose. Reflective–dialogic classroom climate was positively and significantly associated with metacognitive awareness, emotional regulation, and academic purpose, suggesting that classrooms characterized by reflective and dialogic practices tend to be linked with more adaptive cognitive and motivational outcomes at the student level. Metacognitive awareness exhibited a strong positive correlation with academic purpose, indicating that students who are more aware of and able to regulate their cognitive processes also tend to report a clearer sense of direction and meaning in their academic pursuits. Emotional regulation was positively related to both metacognitive awareness and academic purpose, highlighting its relevance in students’ cognitive and motivational functioning. Demographic variables, including age, gender, and grade level, showed weak or non-significant correlations with the focal study variables, suggesting limited confounding influence. Overall, the pattern of correlations provides preliminary support for the proposed multilevel framework while indicating no concerns regarding multicollinearity.

**Table 4:** Descriptive statistics and correlations.

Variables	Mean	SD	1	2	3	4	5	6	7
1. Reflective–dialogic classroom climate	3.61	0.42	—						
2. Metacognitive awareness	3.55	0.58	0.38**	—					
3. Emotional regulation	3.48	0.61	0.29**	0.46**	—				
4. Academic purpose	3.70	0.55	0.41**	0.52**	0.48**	—			
5. Age	15.34	1.12	0.03	0.06	0.04	0.08	—		
6. Gender	—	—	−0.04	0.02	−0.01	0.03	0.01	—	
7. Grade level	2.41	0.64	0.05	0.07	0.06	0.09	0.62**	−0.02	—

Reflective–dialogic classroom climate represents classroom-level aggregated perceptions. All other variables are measured at the individual level; Gender was coded as 0 = female and 1 = male; **p < 0.01.

Table 5 reports the results for convergent and discriminant validity. All constructs demonstrated satisfactory convergent validity, as indicated by composite reliability values exceeding the recommended threshold of 0.70 and average variance extracted (AVE) values above 0.50. Cronbach’s alpha coefficients further supported internal consistency reliability for all constructs. Discriminant validity was established based on multiple criteria. First, for each construct, the square root of AVE exceeded the corresponding inter-construct correlations, satisfying the Fornell–Larcker criterion. Second, the AVE values for all constructs were greater than both the maximum shared variance (MSV) and the average shared variance (ASV), providing additional evidence that the constructs are empirically distinct. Collectively, these results indicate that the measurement model exhibits adequate reliability as well as convergent and discriminant validity, supporting the suitability of the constructs for subsequent multilevel analyses.

**Table 5:** Discriminant and convergent validity.

Construct	α	CR	AVE	MSV	ASV	1	2	3	4
1. Reflective–Dialogic Classroom Climate	0.89	0.92	0.55	0.17	0.13	0.74			
2. Metacognitive Awareness	0.90	0.93	0.60	0.27	0.21	0.38	0.77		
3. Emotional Regulation	0.88	0.91	0.57	0.23	0.18	0.29	0.46	0.75	
4. Academic Purpose	0.91	0.93	0.62	0.27	0.22	0.41	0.52	0.48	0.79

Diagonal elements represent the square root of AVE (√AVE). Off-diagonal elements are construct correlations.

Table 6 presents the confirmatory factor analysis results. The hypothesized four-factor measurement model demonstrated good fit to the data (χ^2^ = 512.38, df = 224, χ^2^/df = 2.29, CFI = 0.952, TLI = 0.944, RMSEA = 0.056, SRMR = 0.043) and outperformed the alternative three-factor, two-factor, and one-factor models, all of which exhibited substantially poorer model fit. These findings support the distinctiveness of reflective–dialogic classroom climate, metacognitive awareness, emotional regulation, and academic purpose, providing further evidence for the adequacy of the measurement model.

**Table 6:** Confirmatory factor analysis model comparison.

Measurement model	χ^2^	df	χ^2^/df	CFI	TLI	RMSEA	SRMR
Four-factor model (reflective–dialogic classroom climate, metacognitive awareness, emotional regulation, academic purpose)	512.38	224	2.29	0.952	0.944	0.056	0.043
Three-factor model (classroom climate; metacognitive awareness + emotional regulation; academic purpose)	873.62	227	3.85	0.892	0.874	0.084	0.071
Two-factor model (classroom climate; remaining constructs combined)	1264.90	229	5.52	0.821	0.793	0.112	0.096
One-factor model (all constructs combined)	1987.43	230	8.64	0.702	0.661	0.158	0.142

CFI, comparative fit index; TLI, Tucker–Lewis index; RMSEA, root mean square error of approximation; SRMR, standardized root mean square residual.

Table 7 presents the multilevel structural path estimates derived from the Mplus analysis. The final multilevel structural model demonstrated satisfactory fit to the data, with an SRMR_within_ value of 0.041 and an SRMR_between_ value of 0.056, indicating acceptable residual fit at both the within-classroom and between-classroom levels. At the between-classroom level, reflective–dialogic classroom climate was positively and significantly associated with metacognitive awareness (*β* = 0.32, SE = 0.07, t = 4.57, *p* = 0.000) and academic purpose (β = 0.22, SE = 0.06, t = 3.67, p = 0.000), indicating that classrooms characterized by stronger reflective and dialogic practices were linked with higher levels of students’ cognitive self-regulation and academic purpose.

**Table 7:** Multilevel structural model results.

Path	β	SE	t	*p*	Effect Size (f^2^)
Level 2 (between-classroom effects)					
Reflective–dialogic classroom climate → metacognitive awareness	0.32	0.07	4.57	< 0.001	0.12
Reflective–dialogic classroom climate → academic purpose	0.22	0.06	3.67	< 0.001	0.07
Cross-level moderation (Stage 1)					
Classroom climate × emotional regulation → metacognitive awareness	0.14	0.04	3.50	0.001	0.03
Level 1 (within-classroom effects)					
Metacognitive awareness → academic purpose	0.38	0.05	7.60	< 0.001	0.18
Emotional regulation → academic purpose	0.27	0.04	6.75	< 0.001	0.10
Moderation (Stage 2)					
Metacognitive awareness × emotional regulation → academic purpose	0.12	0.03	4.00	< 0.001	0.03
Explained variance					
Metacognitive awareness (R^2^)	0.31				
Academic purpose (R^2^)	0.48				
SRMR_within_	0.041				
SRMR_between_	0.056				

β, standardized path coefficient; SE, standard error. Level-1 predictors were group-mean centered, and the Level-2 predictor was grand-mean centered.

**Figure 3:**
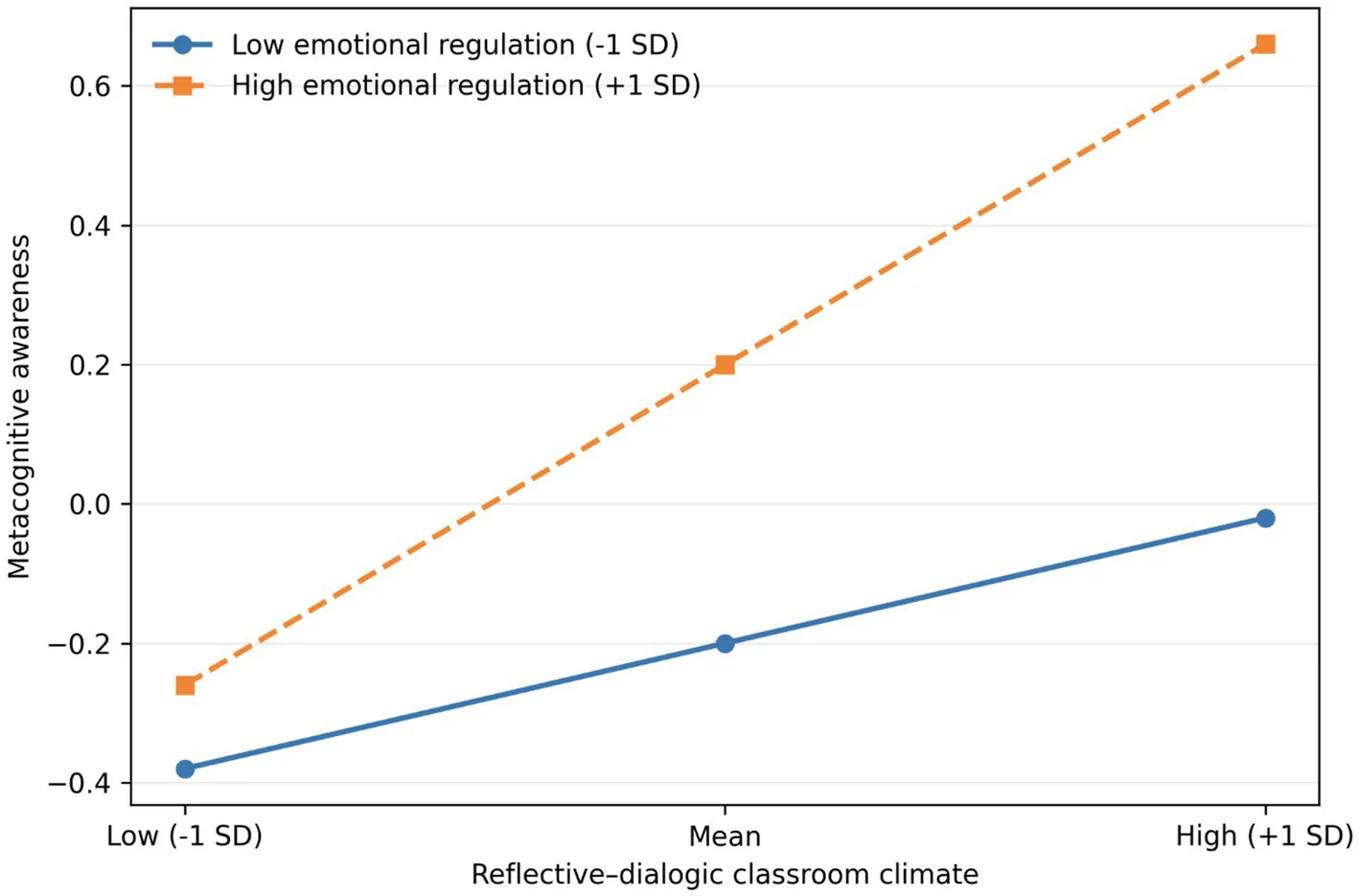
Interaction between classroom climate and emotional regulation.

A significant cross-level interaction between reflective–dialogic classroom climate and emotional regulation emerged for metacognitive awareness (*β* = 0.14, SE = 0.04, t = 3.50, *p* = 0.001), suggesting that the positive association of classroom climate on students’ metacognitive awareness was stronger among students with higher emotional regulation. This interaction is illustrated in Figure 3, and the simple slope analysis confirmed that the positive association between classroom climate and metacognitive awareness was stronger under high emotional regulation than under low emotional regulation.

**Figure 4:**
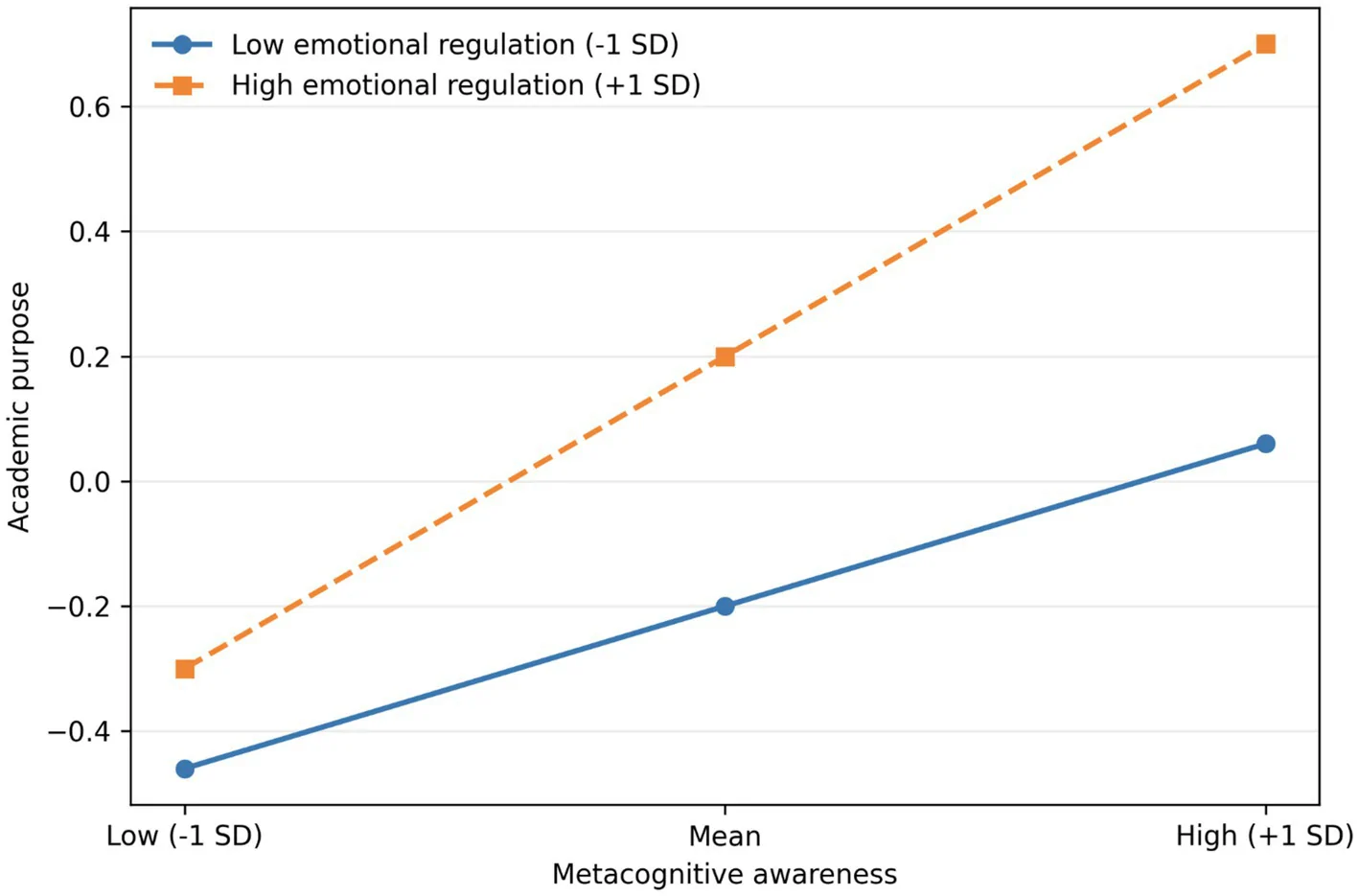
Interaction between metacognitive awareness and emotional regulation.

At the individual level, metacognitive awareness was positively related to academic purpose (*β* = 0.38, SE = 0.05, t = 7.60, *p* = 0.000), and emotional regulation also demonstrated a significant direct association with academic purpose (*β* = 0.27, SE = 0.04, t = 6.75, *p* = 0.000). In addition, the interaction between metacognitive awareness and emotional regulation was statistically significant (*β* = 0.12, SE = 0.03, t = 4.00, *p* = 0.000), indicating that emotional regulation strengthened the positive relationship between metacognitive awareness and academic purpose. Figure 4 further illustrates this interaction, with the simple slope analysis showing that the positive relationship between metacognitive awareness and academic purpose became stronger as emotional regulation increased.

Table 8 presents the indirect and conditional indirect effects. Consistent with a dual-stage moderated mediation framework, the indirect effect of reflective–dialogic classroom climate on academic purpose through metacognitive awareness was significant (*β* = 0.12, SE = 0.03, *p* = 0.000). Conditional indirect effect estimates further showed that this indirect relationship was stronger at higher levels of emotional regulation (*β* = 0.18, SE = 0.04, *p* = 0.000) than at lower levels of emotional regulation (*β* = 0.07, SE = 0.03, *p* = 0.020), supporting the proposed multilevel moderated mediation model.

**Table 8:** Indirect and conditional indirect effects.

Effect	Effect (β)	Boot SE	95% CI	*p*
Indirect effect: Reflective–Dialogic Classroom Climate → Metacognitive Awareness → Academic Purpose	0.12	0.03	[0.06, 0.18]	< 0.001
Conditional indirect effect (High Emotional Regulation; +1 SD)	0.18	0.04	[0.10, 0.26]	< 0.001
Conditional indirect effect (Low Emotional Regulation; −1 SD)	0.07	0.03	[0.01, 0.13]	0.020
Index of moderated mediation	0.05	0.02	[0.02, 0.09]	0.003

Bootstrapped confidence intervals were estimated using 5,000 bootstrap resamples. An indirect effect is considered statistically significant when the 95% confidence interval does not include zero.

## 7 Discussion

The present study advances understanding of adolescents’ academic purpose by demonstrating how classroom-level instructional climate and individual-level self-regulatory processes jointly operate within a multilevel, dual-stage moderated mediation framework. The findings indicate that reflective–dialogic classroom climate is directly and indirectly associated with academic purpose, underscoring the importance of learning environments that encourage reflection, dialogue, and cognitive articulation. This pattern suggests that reflective–dialogic classrooms create opportunities for students to question assumptions, articulate reasoning, and evaluate alternative perspectives, may thereby be associated with deeper self-regulatory engagement rather than merely promoting participation (Brestovanský and Sopoliga, 2025; Stewart and Reinders, 2023). Importantly, the positive association between classroom climate and metacognitive awareness was contingent upon students’ emotional regulation capacity, suggesting that emotionally regulated students are better positioned to respond to contextual learning resources with greater metacognitive awareness. One possible explanation is that emotionally regulated students are better able to manage the uncertainty, cognitive conflict, and evaluative demands inherent in reflective classroom interactions, allowing greater attentional resources to be directed toward monitoring and regulating their own thinking (De Neve et al., 2023; Vistorte et al., 2024). In contrast, students with weaker emotional regulation may allocate more cognitive resources to managing stress or frustration, thereby reducing the benefits derived from the same classroom environment. This cross-level moderation highlights that classroom practices alone are insufficient; rather, their associations with student outcomes may vary according to students’ emotional readiness to engage with reflective and dialogic demands. At the individual level, metacognitive awareness emerged as a central mechanism accounting for the association between classroom climate and academic purpose, aligning with self-regulated learning perspectives that emphasize cognition–emotion integration in purposeful learning. This finding suggests that classroom experiences become meaningful for adolescents not simply because they occur in supportive environments, but because such environments encourage learners to monitor, evaluate, and adapt their learning processes, thereby connecting everyday academic activities with broader educational goals (Zimmerman, 2013; Chandra Handa, 2023). The moderation of the metacognitive awareness–academic purpose relationship by emotional regulation further indicates that cognitive insight translates into academic purpose more strongly when students can effectively manage emotional responses to academic challenges. Students with stronger emotional regulation may be more likely to sustain effort, reinterpret setbacks constructively, and show a stronger association between metacognitive awareness and academic purpose (De Neve et al., 2023; Vistorte et al., 2024). Collectively, the conditional indirect effects demonstrate that emotional regulation functions as a critical boundary condition across both stages of the model, qualifying the indirect association between classroom climate and academic purpose through metacognitive processes. Rather than operating only as an independent motivational resource, emotional regulation appears to be associated with students’ greater use of classroom learning opportunities by enabling them to benefit more fully from reflective experiences and metacognitive insight. These findings contribute to theory by integrating classroom climate research with self-regulatory and emotional frameworks, and by illustrating that the development of academic purpose in adolescence is best understood as a context-sensitive process in which instructional environments and individual regulatory capacities dynamically interact. Accordingly, the findings extend self-regulated learning theory by showing that self-regulation is not solely an internal learner process but is activated by classroom-level affordances and conditioned by students’ emotional regulatory capacity (Zimmerman, 2013; Allen and Vella-Brodrick, 2018).

### 7.1 Research implications

This study makes several important theoretical contributions to the literature on adolescent learning, classroom climate, and self-regulatory development by advancing a multilevel, process-oriented understanding of academic purpose. First, by conceptualizing reflective–dialogic classroom climate as a Level-2 contextual resource, the findings extend classroom climate research beyond direct outcome models and position instructional environments as contextual resources associated with students’ internal self-regulatory mechanisms. Prior work has acknowledged the importance of dialogic and reflective pedagogies for engagement and learning outcomes (Boyd et al., 2022; Cui and Teo, 2021; Hofmann et al., 2021), yet empirical evidence explaining how such climates are related to deeper motivational orientations has remained limited. The present study addresses this gap by showing that reflective–dialogic classroom climate is indirectly associated with academic purpose through students’ metacognitive awareness, thereby integrating classroom discourse theory with self-regulated learning frameworks (Brenner, 2022; Zimmerman, 2013).

Second, the study advances self-regulated learning theory by explicitly incorporating emotional regulation as a boundary condition operating at multiple stages of the regulatory process. While cognitive and metacognitive processes have long been central to models of self-regulated learning, emotional regulation has often been treated as an ancillary or distal factor. The present findings challenge this separation by showing that emotional regulation moderates both the contextual association between reflective–dialogic classroom climate and metacognitive awareness and the association between metacognitive awareness and academic purpose. This dual-stage moderation suggests that metacognitive processes are not uniformly activated or equally relevant across learners; rather, their associations with classroom conditions and motivational outcomes depend on students’ capacity to manage the emotional demands associated with reflective and dialogic learning. In doing so, the study contributes to emerging integrative models that emphasize the interdependence of cognitive, emotional, and motivational regulation in academic development (Efklides and Schwartz, 2024; Merkebu et al., 2023; Zheng et al., 2023).

More broadly, these findings contribute to the further development of self-regulated learning theory in three respects. First, they support a context-sensitive view of self-regulation by indicating that regulatory processes are not generated solely within the learner but may be associated with shared instructional affordances at the classroom level. Second, they suggest that emotional regulation should be conceptualized as an integral component of the self-regulatory system rather than as a peripheral personal characteristic because it conditions both the association between classroom climate and metacognitive awareness and the association between metacognitive awareness and academic purpose. Third, the conditional indirect pattern indicates that self-regulated learning may be better understood as a multilevel process in which classroom-level opportunities, individual metacognitive monitoring, and emotional regulatory resources operate jointly. Accordingly, the study extends conventional learner-centered accounts of self-regulated learning toward a more integrative framework that explains how contextual and individual regulatory processes are jointly associated with the development of future-oriented academic meaning.

Third, by adopting a multilevel perspective, the study contributes to theory on purpose development in adolescence. Research on academic purpose has predominantly focused on individual traits, values, and goal orientations (Joshanloo, 2024; Kim et al., 2022), often overlooking the role of proximal educational contexts. The present findings indicate that academic purpose is not solely an internally generated construct but may reflect ongoing interactions between classroom environments and students’ regulatory capacities. The identification of a conditional indirect association between classroom climate and academic purpose provides theoretical support for viewing purpose as an emergent, context-sensitive construct rather than a static motivational disposition. This perspective aligns with developmental and ecological theories that emphasize the co-construction of meaning through person–environment interactions during adolescence (Baoguo and Xiaobing, 2025; McCarthy, 2022).

The findings of this study carry several important implications for educational practice, curriculum design, and teacher professional development. First, the results emphasize the value of cultivating reflective–dialogic classroom climates as a means of supporting students’ academic purpose. Educators and school leaders should recognize that instructional practices encouraging reflection, explanation, and peer dialogue may do more than enhance surface engagement; they may also create conditions associated with students’ metacognitive development and long-term academic meaning. Professional development initiatives should therefore prioritize pedagogical strategies that integrate structured reflection, dialogic questioning, and collaborative sense-making into everyday classroom practice, rather than treating such activities as supplementary or enrichment-based.

Second, the dual moderating role of emotional regulation highlights the need for greater alignment between academic instruction and social–emotional learning initiatives. The findings suggest that reflective and dialogic pedagogies may be more strongly associated with beneficial outcomes among students who possess sufficient emotional regulation skills to manage the cognitive and interpersonal demands of such environments. Consequently, schools should avoid assuming that reflective instructional designs will benefit all students equally. Instead, emotional regulation skills—such as emotional awareness, reappraisal, and stress management—should be explicitly cultivated alongside metacognitive and academic instruction. Integrating emotional regulation training into classroom routines may strengthen students’ capacity to engage productively with reflective learning opportunities and may enhance the motivational value associated with dialogic teaching.

Third, the results have implications for differentiated instruction and equity-oriented pedagogy. Because emotional regulation functions as a boundary condition across multiple stages of the learning process, students with lower regulatory capacities may be less able to benefit from highly reflective and dialogic classrooms when adequate support is not provided. Educators should therefore consider scaffolding reflective tasks, offering emotional support strategies, and creating psychologically safe discussion environments to ensure that all students have opportunities to benefit from dialogic instruction. Such practices may be particularly important in adolescent classrooms, where emotional variability and sensitivity to peer evaluation are pronounced.

Finally, at the policy level, the findings suggest that evaluations of instructional quality should move beyond narrow performance indicators and incorporate indicators of classroom climate and self-regulatory development. Educational policies that emphasize test performance without attending to reflective learning environments and emotional competencies risk overlooking foundational processes associated with sustained academic purpose. By foregrounding the joint roles of classroom climate, metacognitive awareness, and emotional regulation, the present study provides a framework for designing learning environments that support not only academic achievement but also students’ long-term engagement and sense of meaning in education.

### 7.2 Limitations and future research

Despite its contributions, the present study is subject to several limitations that should be acknowledged and addressed in future research. First, the cross-sectional nature of the data constrains causal inference. Although the multilevel and moderated mediation analyses are theoretically grounded and statistically robust, the temporal ordering of classroom climate, metacognitive awareness, emotional regulation, and academic purpose cannot be definitively established. Moreover, because all variables were measured during the same data-collection period, the observed associations may partly reflect contemporaneous response tendencies, transient mood states, consistency motives, or respondents’ implicit theories about how the constructs should relate. The simultaneous measurement of the predictor, mediator, moderator, and outcome also prevents examination of whether changes in classroom climate precede changes in metacognitive awareness and academic purpose. Future studies would benefit from longitudinal or time-lagged designs to examine how reflective–dialogic classroom climate is associated with changes in metacognitive and emotional regulation capacities over time and how these processes are subsequently related to trajectories of academic purpose across adolescence.

Second, the reliance on self-reported measures may introduce common method variance and social desirability bias. While self-reports are appropriate for capturing internal cognitive and emotional processes, the use of a common measurement source and format may have inflated some of the observed relationships, even though the statistical assessments did not indicate substantial common method bias. Such diagnostic tests cannot completely exclude bias arising from shared measurement conditions. Future research could strengthen validity by incorporating multi-source data, such as teacher reports of classroom practices, observational assessments of dialogic instruction, or performance-based measures of metacognitive regulation.

Third, although reflective–dialogic classroom climate was modeled at the classroom level, the aggregated measure was derived from students’ individual perceptions rather than from independent observations of classroom practices. Aggregation assumes that students within the same classroom share a sufficiently common experience and that the resulting classroom mean represents a collective contextual property. Although the observed within-class agreement and intraclass correlations supported aggregation, students may nevertheless differ in how they interpret and evaluate the same instructional environment. The classroom-level score may therefore conceal meaningful within-class heterogeneity and may partly reflect shared perceptual tendencies rather than objective characteristics of classroom instruction. In addition, unequal classroom sizes can affect the precision of aggregated means, with estimates from smaller classrooms potentially being less stable. The study also did not explicitly account for variability in teacher characteristics, instructional experience, or pedagogical training, which may be associated with the enactment of reflective and dialogic practices. Future research could extend the present model by incorporating teacher-level predictors, independent classroom observations, teacher-reported climate measures, or random slopes to examine whether the strength of the observed associations varies across instructional contexts or educator profiles.

Fourth, the sample was drawn from a specific educational and cultural context, which may limit the generalizability of the findings. Cultural norms regarding classroom dialogue, emotional expression, and student–teacher interaction may shape how reflective and dialogic practices are experienced by students. Future research should examine the proposed multilevel framework across diverse educational systems and cultural settings to assess its robustness and boundary conditions.

Finally, while emotional regulation was examined as a moderator at two stages of the model, other individual differences—such as academic self-efficacy, motivational orientation, or peer sensitivity—may also condition the associations between reflective classroom environments, metacognitive awareness, and academic purpose. Future studies could explore more complex regulatory profiles or person-centered approaches to identify subgroups of students for whom dialogic and reflective pedagogies are differentially associated with academic outcomes.

## 8 Conclusion

The present study advances understanding of adolescents’ academic purpose by integrating classroom-level instructional climate with individual cognitive and emotional self-regulatory processes within a multilevel, dual-stage moderated mediation framework. By highlighting the joint associations of reflective–dialogic classroom climate, metacognitive awareness, and emotional regulation with academic purpose, the findings contribute to classroom climate research, self-regulated learning theory, and purpose development while providing a multilevel framework for future research on adolescents’ motivational development.

## Data Availability

The original contributions presented in the study are included in the article/supplementary material, further inquiries can be directed to the corresponding author.
